# Developing and Evaluating a New Consultation Service for Complex Psychosis in Kent, United Kingdom

**DOI:** 10.1192/bjo.2026.11576

**Published:** 2026-06-30

**Authors:** Su Ying Yeoh, Eromona Whiskey, Aderopo Adelola, Suresh Thapaliys, Sukhwinder Shergill

**Affiliations:** 1 Kent and Medway Mental Health NHS Trust, Canterbury, United Kingdom; 2 Kent and Medway Medical School, Canterbury, United Kingdom; 3 South London and Maudsley NHS Trust, London, United Kingdom; 4 Essex Partnership University NHS Foundation Trust, Chelmsford, United Kingdom; 5 Kings College London, London, United Kingdom

## Abstract

**Aims:**

Complex psychosis, characterised by severe, treatment-resistant psychosis with associated functional impairment, is one of the most challenging conditions to manage in psychiatric practice. Evidence-based guidelines recommend clozapine for treatment-resistant psychosis, though in practice less than 1/3 of potentially eligible patients receive clozapine in a timely manner. The Complex Psychosis Service (CPS) was established in Kent in 2022, with the goal of providing a specialist multi-disciplinary opinion to clinicians for the management of patients with complex psychosis.

This paper aims to describe our model for developing and implementing this new consultation service for complex psychosis in Kent and Medway Mental Health NHS Trust. We aim to describe the clinical characteristics of individuals referred to CPS, treatment recommendations made, rates of uptake, and staff experiences of the service.

**Methods:**

Retrospective data was collected on the number of referrals to CPS from its inception in December 2022 to September 2024. Data was collected on several measures including primary psychiatric diagnosis, comorbid mental disorders and prior antipsychotic treatment, including whether clozapine had been trialled in the past. Questionnaire data was collected from referrers, and qualitative experiences explored through semi-structured interviews with six referrers and discussions with the CPS team.

**Results:**

62 referrals were made to CPS during this time period. The most common primary diagnoses were treatment-resistant schizophrenia (n=29) and schizoaffective disorder (n=19), and 26 had another comorbid mental disorder, most frequently mood disorder (n=7) and personality disorder (n=5). Patients had a mean of 5.7 prior inpatient admissions and average of 6.2 antipsychotic trials. 18 patients had never tried clozapine despite treatment-resistance. CPS recommendations proposed trials of clozapine (n=12), augmentation with other psychotropics (n=16), ECT augmenting clozapine (n=5), and non-pharmacological interventions including psychology and occupational therapy.

56% of referrers implemented the recommendations and 90% reported high levels of satisfaction with the service provided by CPS. However, some referrers felt limited by CPS often not reviewing patients face-to-face. Qualitative data indicated that referrers valued the specialist input and expertise, and Psychiatry trainee doctors felt that working in CPS offered a rich educational experience.

**Conclusion:**

Individuals referred to CPS primarily had schizophrenia-spectrum illness with treatment-resistance, with significant burden from untreated symptoms. Input from the CPS multi-disciplinary team was well-received by referrers, and there were high levels of satisfaction both from referrers and CPS clinicians. Future directions to expand CPS would include specialist psychology input, and potentially inpatient beds on a specialist unit to increase the uptake of treatment recommendations.

